# Ovarian Hyperstimulation syndrome combined with hypothyroidism: a comprehensive review

**DOI:** 10.1186/s13048-024-01406-3

**Published:** 2024-05-09

**Authors:** Jing Zhou, Yu Chen, Lijing Bai, Wei Zhou, Haiyan Yang, Yang Chen, Li Chen, Renjie Lu, Lingmin Hu, Shuxian Wang

**Affiliations:** 1 Department of Reproduction, Changzhou Maternity and Child Health Care Hospital, Changzhou Medical Center, Nanjing Medical University, Changzhou, 213000 Jiangsu China; 2 Changzhou Third People’s Hospital, Changzhou Medical Center, Nanjing Medical University, Changzhou, 213000 Jiangsu China; 3 Changzhou Institute for Advanced Study of Public Health, Nanjing Medical University, Nanjing, 210000 Jiangsu China

**Keywords:** Ovarian hyperstimulation syndrome, Hypothyroidism, Case report

## Abstract

Ovarian Hyperstimulation Syndrome (OHSS) is a systemic condition marked by the enlargement of the ovaries and heightened vascular permeability. And hypothyroidism (HT) emerges as a potential risk factor for OHSS occurrence. This review presented a comprehensive summary of pertinent case reports involving patients diagnosed with both HT and OHSS. Detailed exploration was conducted into their clinical presentations, diagnostic methodologies, and treatment modalities. Additionally, the review delved into potential interaction mechanisms between HT and OHSS, encompassing various aspects including hormone levels. Moreover, management strategies for mitigating the risk of OHSS in HT patients were thoroughly reviewed and the importance of monitoring thyroid function in those experiencing OHSS was emphasized. This review indicated that the association between HT and OHSS, underscoring its multifaceted complexity. It could accentuate the ongoing necessity for rigorous research and clinical refinement to deepen our comprehension of this association and to bolster diagnostic and therapeutic methodologies for optimal patient care. In conclusion, this review offered valuable insights for future research directions and clinical practices for patients afflicted with OHSS and HT.

## Introduction

Ovarian Hyperstimulation Syndrome (OHSS) is a systemic disorder characterized by ovarian enlargement and increased vascular permeability [1–3]. In this review, OHSS occurring in patients following controlled ovarian hyperstimulation (COH) with exogenous gonadotropins or a few other drugs is defined as the iatrogenic form of ovarian hyperstimulation syndrome (iOHSS) [2–4]. Additionally, OHSS that occurs in pregnant women between the eighth and fourteenth weeks of pregnancy is termed as spontaneous ovarian hyperstimulation syndrome (sOHSS) [3–5]. Furthermore, some non-pregnant patients may also experience sOHSS. Therefore, sOHSS in this review is defined as OHSS that occurs without the use of any exogenous drugs for COH [6].

The incidence rates of OHSS vary globally. Although OHSS can occur in women with singleton or multiple pregnancies, HT, and pituitary adenomas, with or without pregnancy in very rare cases [7, 8], the overall incidence rate of OHSS accounts for approximately 33% of all cycles of in vitro fertilization (IVF) cycles worldwide [9]. In China, the incidence rate of moderate and severe OHSS in women of reproductive age was 1.14% [10]. To sum up, the prevalence of OHSS related to assisted reproductive technology (ART) remains relatively high among females of reproductive age, with potential life-threatening risks. Accurately identifying the risk of OHSS, along with implementing effective prevention and targeted treatments, may help reduce its incidence rate in women of reproductive age and prevent its progression into fatal OHSS. Therefore, there is an urgent need for in-depth attention towards the prevention and treatment of OHSS to safeguard women’s reproductive health.

Potential risk factors for OHSS include age less than 30 years old, slender physique, polycystic ovarian syndrome (PCOS), supplementation of exogenous or endogenous human chorionic gonadotropin (HCG) during the luteal phase, use of gonadotropin-releasing hormone (GnRH) for ovulation triggering, serum high concentration of estradiol(E_2_) (> 4000 pg/ml), serum anti-Müllerian hormone (AMH) concentration more than 3.36 ng/ml, antral follicle count (AFC) more than 8, acquisition of a large number of follicles during ovulation triggering, count of follicles with a diameter around 11 mm (range from 8 mm to 12 mm) on the day of ovulation triggering over 14, hyperprolactinemia, oligomenorrhea, anovulatory infertility, HT, and a previous history of OHSS [4, 8, 11–14]. However, research on the association between HT and OHSS is limited. Therefore, this review focuses on OHSS related to HT, providing guidance on future research directions and preventive measures based on existing studies.

HT is defined as a condition characterized by thyroid hormone levels in the serum being below normal [15]. Evidence from a study indicates that HT might be a risk factor for the occurrence of OHSS [16]. The clinical parameters of thyroid function can be used to predict the risk of developing OHSS, such as thyroid-stimulating hormone (TSH), free thyroxine (FT4), and thyroid peroxidase antibodies (TPO-Ab), enabling targeted preventive measures by healthcare professionals [16]. As an essential medical intervention in ART, COH might further increase the risk of OHSS in women with HT [17]. A retrospective study has confirmed that in the population of non-polycystic ovarian syndrome (NPCOS), women with HT that undergo COH have a fivefold increased risk of OHSS compared to those without HT [18]. This is attributed to the potential of COH to further decrease serum FT4 levels and increase serum TSH levels, exacerbating HT [19, 20]. Additionally, some research findings have suggested that elevated serum TSH levels during or after COH might be associated with the excessive use of ovulation-inducing drugs [21]. Through a prospective cohort study, it has been noted that gonadotrophin-releasing hormone analogue (GnRH-α), a type of ovulation-inducing drug, significantly increases serum TSH concentrations [21]. Therefore, women with HT may have a higher risk of OHSS after COH [16].

Currently, there is no definitive conclusion regarding whether COH contributes to structural thyroid abnormalities. In a retrospective study, it was found that women with initially normal thyroid function, regardless of whether OHSS occurred, experienced varying degrees of increased serum TSH levels after COH [22]. However, the elevated state of serum TSH levels caused by COH is not persistent in these patients. Consequently, these women do not ultimately develop subclinical hypothyroidism (SHT) or HT. This may be attributed to notable surge in serum E_2_ levels after COH, leading to an elevation in serum thyroxine-binding globulin (TBG) levels. The increased TBG binding with excess FT4 may result in a transient elevation of serum TSH. However, serum TSH levels gradually decrease as the patients achieve successful pregnancy, which is attributed to the elevated serum HCG levels competitively binding to thyroid-stimulating hormone receptors (TSH-R), increasing serum FT4 levels and lowering serum TSH levels through negative feedback [23, 24]. Conversely, another prospective cohort study confirmed that COH may induce SHT in patients with initially normal thyroid function [21].

Nevertheless, the exact factors contributing to the varied outcomes in the studies are still not clear. Existing studies did not delve into the mechanisms underlying the association between HT and OHSS, nor did they explore the specific roles and mechanisms of COH in the intricate relationship between HT and OHSS. Therefore, it is necessary to conduct a review of case reports related to HT combined with OHSS to guide subsequent research on mechanisms.

## Review of case reports on OHSS combined with HT

After searching the literatures spanning from January 1, 1980, to January 1, 2024 from the PubMed, Google Scholar, and Web of Science, a total of 18 case reports were collected, detailing sOHSS combined with HT in non-pregnant women. Additionally, 13 case reports were identified for pregnant women experiencing sOHSS concomitant with HT. Furthermore, there were 5 case reports related to iOHSS combined with HT in the women undergoing COH.

### sOHSS combined with HT in non-pregnant women

Through the analysis of 18 case reports involving non-pregnant women with HT and OHSS [6, 8, 25–40], it was observed that the average age of the patients involved in these case reports was 19 years old (M ± SD: 19.08 ± 4.32). All patients exhibited elevated serum TSH levels, reduced serum FT4 concentrations, and normal to low serum luteinizing hormone (LH) and follicle stimulating hormone (FSH) levels. Their clinical manifestations were similar, characterized by ascites and bilateral massive ovarian cysts. Following conservative treatment, involving levothyroxine (LT4) replacement therapy at a dose ranging from 0.05 to 0.2 mg per day, their ovarian sizes normalized within 1 to 6 months. Some more information about these reports can be seen in Table 1.

**Table 1:** Case reports of sOHSS combined with HT in non-pregnant women

No	Age	TSH	FT4	ATPO	T_3_RU	FT3	LH	FSH	E_2_	Others	Ultrasonography Report (bilateral ovarian size)	Treatment	The duration of returning to normal
Rotmensch and Scommegna [25]	21	>25μU/ ml	0.5 μg /dL	–	21.4		6.2 mIU/ml	19.2 mIU /ml	1303 pg/ml	Cholesterol 243 mg/dL	maximum diameter: 10 cm and 13.8 cm；	LT4 replacement treatment	4 weeks
Van Voorhis, Neff [35]	26	> 50 IU/L	–	–	–	–	0.7 IU/L	15.7 IU/L	80 pg/mL	–	–	LT4 replacement treatment	3 months
Chen, Chen [28]	20	190.42uIU/ml	0.15 ng/dL	–		29.8 ng/dL	7.2 mIU/ml	18.4 mIU /ml	1100 pg/ml	–	8.1 × 4.7 cm; 7.4 × 6.1 cm	LT4 replacement treatment	2.5 months
Taher, Ghariabeh [29]	22	> 100 mU/L	< 5 pmol/L	–	–	–	12.6 IU/L	9.8 IU/L	150.9 pmol/l	CA125 93 U/ml	9 × 12 cm; 6 × 4 cm	LT4 replacement treatment	1 year
Guvenal, Guvenal [34]	28	100,000mIU/ ml	0.01 ng/dl	–	–	0.84 pg/ml	–	–	–	CA125 53.3 U/ml	8.2 × 7.5× 5 cm; 7.5 × 6.5 × 4.0 cm	LT4 replacement treatment	3 months
Hedayati Emami, Molaei Langroudi [26]	15	> 100 mIU/L	Low	290 U/ml	31.2	–	–	–	–	Cholesterol 290 mg/dL	15 × 7.5 cm; 13 × 7 cm	LT4 replacement treatment	4 months
	14.5	72.5 mIU/L	Low	439 IU/ml	25.9	–	–	–	–	–	11 × 6.5 cm; 11.8 × 5.8 cm	LT4 replacement treatment	3 months
Langroudi, Amlashi [27]	15	> 100 mIU/l	Low	290 U/ml	31.2%	–	–	–	–	Cholesterol 290 mg/dl	15 × 7.5 × 6.2 cm; 13 × 7 × 6.8 cm	LT4 replacement treatment	4 months
Kanza, Gagnon [6]	19	> 100 mU/L	Low	–	–	–	–	Normal	6000 pmol/l	CA125 87 kU/L	10 × 6 × 10 cm; 9 × 8 × 8 cm.	LT4 replacement treatment	4 months
Katulande, Kariyawasam [36]	23	>100mIU/l	Low	–	–	–	1.0 IU/l	6.1 IU/l	4095 μg/l	–	maximum diameter: 10 cm	LT4 replacement treatment	4 months
Erol, Erol [37]	18^a^	100 lU/mL	4.5 pmol/L	–	–	–	9.2 IU/L	6.5 IU/L	4.000 mIU/mL	–	Enlarged	LT4 replacement treatment	4 months
	18^a^	20 lU/mL	15 pmol/L	–	–	–	–	–	10.000 mIU/mL	–	Enlarged	LT4 replacement treatment	6 months
Singh, Singh [38]	18	102 μlU/ml	Low	–	–	Low	–	–	–	–	15.22 × 8.26 cm; 11.82 × 6.24 cm.	LT4 replacement treatment	4 months
Ilanchezhian, Mohan [33]	25	>150mIU/ ml	Low	–	–	–	Low	12.84 mIU /ml	848.93 pg/ ml	–	7 × 8.7 × 10.7 cm；7.3 × 10.3 × 10.2 cm	LT4 replacement treatment	1.5 months
Putta, John [31]	15	750mIU/ ml	Low	–	–	–	–	–	–	CA125 98 U/l	Enlarged	LT4 replacement treatment	3 months
Rajaram, Bhaskaran [32]	19	>150mIU/ l	–	173.8 U	–	–	–	–	–	–	9.5 × 8 cm; 11 × 8 cm	LT4 replacement treatment; surgery	6 months
Kim, Yoon [39]	14	> 1000 μU/mL	Low	–	–	–	–	7.31 mIU/mL	–	CA125 25.87 U/mL	8.9 × 4.6 cm; 5.9 × 8.2 cm	LT4 replacement treatment	6 months
Patel and Nath [40]	22	30.45μlU/ml	–	56.97 IU/ml	–	–	Low	Low	8.87 pg/ ml	–	Volume:59.6 ~ 111.81 cm^3^，Diameter: 1.4 ~ 20 cm	LT4 replacement treatment	4 months
Pail, Bagri [8]	17	486μIU/ ml	Low	–	–	–	–	–	–	–	12 × 19 × 13 cm 10 × 14 × 11 cm	LT4 replacement treatment	3 months
Kaluarachchi, Casather [30]	12	>100mIU/L	Low	(+)	–	–	–	–	–	CA125 387 U/mL	11 × 10.8 cm; 9.5 × 8 cm	LT4 replacement treatment	3 months

^a^represents the same person

Along with elevated serum E_2_ levels, the reason for a patient with HT and sOHSS might be that E_2_ tend to convert into higher active estriol (E_3_) under higher serum E2 concentration, leading to increased release of FSH and sOHSS in one case report [25]. Additionally, this case report has shown that the patient with HT and sOHSS had elevated serum total cholesterol, and low-density lipoprotein cholesterol has been shown to enhance steroid synthesis in human granulosa cells in vitro [25], which could also be seen in other case reports [26, 27]. Therefore, it is speculated that the elevated total cholesterol levels might also play a role in the development of OHSS combined with HT [25]. However, patients combined with sOHSS and HT may be also characteristic of non-elevated serum FSH levels and elevated serum E_2_ concentrations [28]. Elevated serum E_2_ concentrations were found to stimulate increased release of TSH, subsequently activating follicle stimulating hormone receptors (FSH-R) and leading to OHSS [29]. Additionally, despite the presence of hyperprolactinemia in the patient, normalization of serum prolactin levels was observed following LT4 replacement therapy. Consequently, considering the restorability of endocrine function, excessive pharmacological or surgical interventions may be unnecessary. Furthermore, OHSS and hyperprolactinemia may constitute notable features of HT [28].

In some other case reports, elevated serum CA125 levels may be another characteristic of patients combined with OHSS and HT, which is similar to ovarian cancer [6, 29–31]. After initiating exploratory treatment with LT4 replacement therapy, various symptoms have been improved, which prevented misdiagnosis and unnecessary treatment for ovarian cancer [29]. Therefore, the indispensability of evaluating thyroid function parameters should be emphasized when encountering cases of OHSS, which will help prevent inappropriate surgical interventions.

Furthermore, some patients combine with OHSS and HT may be diagnosed with autoimmune thyroid disease. A distinctive feature of this disease is the presence of autoantibodies against thyroid antigens within the patients. Antibodies against the TSH-R predominantly target the α-subunit of the TSH-R (with activating or inhibitory effects). And both TSH-R and FSH-R share a common α-subunit. Therefore, elevated serum TPOAb levels may interact with the FSH-R, leading to the occurrence of OHSS [41].

Based on existing case reports, while the majority of the patients were treated with LT4 conservatively, there were also a small number of cases that underwent surgical intervention [32]. In one case report, even with a daily dose of 0.1 mg LT4, symptoms of the patient were not well controlled. Consequently, surgical removal of some cysts and oral intake of LT4 were initiated. Six months later, the ovarian size of this patient returned to normal [32]. Therefore, conservative LT4 replacement therapy may not be universally effective for HT combined with OHSS. In cases where conservative LT4 replacement therapy proves ineffective, timely surgical cyst removal combined with pharmacological intervention may be necessary [32].

### sOHSS combined with HT in naturally pregnant women

Through the analysis of 13 case reports on HT combined with OHSS in naturally pregnant women [42–54], it has been found that the average age of these patients was 26 years (M ± SD: 26 ± 4.26). Most pregnant women experienced OHSS between the 9th and the 14th weeks of pregnancy. The clinical changes in serum TSH, FT4, LH, and FSH levels in this group were similar to those in non-pregnant women. While the treatment for most pregnant women was similar to that of non-pregnant women, it was evident that the treatment plans for pregnant women, as a special group, were more personalized. Physicians adjusted the dosage of orally administered LT4 based on the gestational week. The time for the normalization of ovarian size varied among them; for some pregnant women, ovarian size could return to normal during pregnancy, while for others, it might only normalize after childbirth. Some more information about these reports can be seen in Table 2.

**Table 2:** Case reports of sOHSS combined with HT in naturally pregnant women

No	Age	Pregnancy	TSH	FT4	FT3	LH	FSH	β-hCG	E_2_ pg/ml	CA125	Ultrasonography Report (bilateral ovarian size)	Treatment	The duration of returning to normal	Successful delivery gestational weeks
Nappi, Di Naro [44]	34	12w	> 350 μU/ml	Low	Low	0.5 mIU/ml	0.6 mIU/ml	12,8461 mIU/ml	9150	–	Diameter:13 cm, Diameter: 11 cm	LT4 replacement treatment	2 weeks	38 weeks
Cardoso, Graça [45]	25	12w	210 mIU/mL	0.2 ng/dL	1.3 pg/mL	–	–	15,890 mIU/mL	–	74.5 U/mL	16 × 15 cm; 16 × 13 cm	LT4 replacement treatment	24 weeks gestation	28 weeks
Borna and Nasery [47]	30	20w	>400μU/mL	0.4 ng/dL	1.1 pg/mL	–	–	–	–	39 U/mL	20 × 16 cm; 16 × 10 cm	LT4 replacement treatment	10 weeks after delivery	38 weeks
Edwards-Silva, Han [42]	30	10w	41.7 mU/L	–	–	–	–	291,206 mIU/mL	–	901 U/mL	10 × 14 × 7 cm; 10 × 12 × 8 cm	LT4 replacement treatment	–	34 weeks
Lussiana, Guani [46]	29	22w	5.92 mU/l	Normal	Normal	Normal	Normal	High	High	–	20 × 11 cm; 16 × 12 cm	–	3 weeks after abortion	abortion
Akbay, Uzunçakmak [48]	21	10w^a^	8.75 IU/mL	–	–	–	–	High	–	146.8 IU/ml	13 × 8 cm	LT4 replacement treatment	3 weeks after delivery	–
	23	12w^a^	2.16 IU/mL	–	–	–	–	High	–	289 IU/ml	1.3 × 7 cm; 11 × 7 cm	LT4 replacement treatment	6 weeks after delivery	38 weeks
Dieterich, Bolz [43]	26^b^	12w	3.73mIU/l	13.9 pmol/l	4.8 pmol/l	–	–	118,665 IU/l	–	–	–	Diuresis; Fluid drainage	–	Termination of pregnancy (15 weeks)
	26^b^	10w	5.51mIU/l	15.3 pmol/l	3.8 pmol/l	–	–	147,688 IU/l	–	–	6 × 7 cm; 5 × 7 cm	LT4 replacement treatment	18 weeks gestation	39 weeks
Delabaere, Tran [49]	23	12w	High	Low	Normal	Normal	Normal	Match	–	–	17 × 10.2 cm; 14.2 × 7 cm	LT4 replacement treatment	3 months	39 weeks
Sridev and Barathan [50]	22	9w	150 μIU/ml	–	–	–	–	181,264.20mIU/ml	–		10 × 8 cm; 8 × 6 cm	LT4 replacement treatment	20 weeks gestation	39 weeks
SEETHAPATHY [51]	20	14w	222mIU/ml	0.07 ng/dl	< 0.26 g/ml	–	–	1,36,776 mIU/ml	–	–	15 × 9 × 13 cm; 12 × 8 × 14.6 cm	LT4 replacement treatment	4 months after abortion	Abortion
Oliveira, Innecco Arêas [52]	32	13w	100 mU/L	0.25 ng/dl	–	–	–	–	–	125 56 U/ml	9.3 × 6.3 × 5.9 cm; 10.6 × 10.0 × 7.1 cm	LT4 replacement treatment	8 months after delivery	37 weeks
Alzebidi, Almushri [53]	27	10w	123mIU/ml	Low	–	< 3 IU/l	11.92mIU/ml	23150mIU/ml	758.93	11 IU/ml	10 × 9.7 × 11.7 cm; 7.5 × 10.5 × 10.3 cm	LT4 replacement treatment	3 months after delivery	–
Guerra, Marado [54]	22	9w	515 IU/mL	0	–	–	–	–	–	1045 U/mL	15 × 14 × 16.6; 15× 8.2 × 18.3 cm	LT4 replacement treatment	3 months after abortion	Termination of pregnancy (10 weeks)

^a,b^represents the same person

Similar to non-pregnant women combined with HT and OHSS, a pregnant woman combined with them could also be characteristic of higher serum CA125 concentration, exhibiting a severe risk of thrombosis [42]. Considering that CA125 can promote thrombus formation by increasing leukocyte and platelet activity [55], it is speculated that a sharp elevation in CA125 levels in pregnant women with HT and OHSS may give rise to concerns about an increased risk of thrombosis. In addition, a mutation in the FSH-R can also cause the occurrence of OHSS and HT. In a case report, a pregnant woman experienced sOHSS combined with HT, resulting from a mutation in the FSH-R (FSH-R D567N) and causing hypersensitivity to HCG and elevated androgen levels [43].

Considering the elevated estrogen levels in the pregnant woman, it was proposed that altered estrogen metabolism might result in inadequate pituitary feedback, leading to increased release of TSH [44]. Excessive TSH levels in ovarian tissue could induce severe cystic reactions in the ovaries, with fluid shifting to the third space, giving rise to OHSS. However, not all pregnant women would successfully deliver at full term after LT4 replacement therapy. One patient delivered prematurely at 28 weeks of gestation after treatment, giving birth to a single fetus [45]. Luckily, due to the premature initiation of LT4 replacement intervention, the newborn demonstrated normal development at the age of 2 [45]. Other patients with mutation in FSH-R could not achieve the control of this disease until terminating the pregnancy [43, 46]. Therefore, unlike sOHSS caused by mutated FSH-R, HT combined with sOHSS can be effectively intervened with LT4 replacement therapy. Early initiation of LT4 replacement therapy seems necessary for pregnant women with HT combined with OHSS.

### iOHSS combined with HT in women

There are fewer case reports related to the women undergoing COH, with five case reports suggesting an average age of 32 years old (M ± SD: 32 ± 4.3) in this group. Most patients undergoing COH experienced OHSS within 1 to 14 days after the procedure, including COH or fresh embryo transfer. All patients showed an increase in serum TSH levels and a decrease in serum FT4 levels. Their clinical symptoms were similar to those described in the aforementioned cases. Analyzing these five case reports, it can be observed that the risk of developing OHSS is higher in the women undergoing COH by exogenous hormones. Therefore, their conservative treatment plans are the most personalized. The personalized drug intervention often begins before COH. Some more information about these reports can be seen in Table 3.

**Table 3:** Case reports of iOHSS combined with HT

No	Age	Pregnancy	Embryo transfer	Protocol of COH	TSH	FT4	FT3	E_2_	β-hCG	Ultrasonography Report (bilateral ovarian size)	Treatment	The duration of returning to normal	Successful delivery gestational weeks
Poppe, Glinoer [22]	37	Yes	Fresh embryo transfer	Antagonis Protocol	41.5mIU/L	7.7 ng/L	–	5.549 ng/L	360 IU/L	Diameter: 10 cm	LT4 replacement treatment	44 days	–
Ghianda, Loconte [56]	28	No	Fresh embryo transfer	–	61.3μU/mL	4.8 pg/ mL	2.54 pg/mL	2651 pg/ml	(−)	–	Cease COH	1 month	–
Skweres, Wójcik [57]	34	Yes	Fresh embryo transfer	Classic long protocol.	5.127 mU/L	19.33 pmol/l	4.22 pmol/L	–	–	–	LT4 replacement treatment	20 weeks gestation	37 weeks
Galvao, Lourenço [58]	27	Yes	Fresh embryo transfer	Antagonis Protocol	28.50μUI/mL	–	–	2854.0 pg/mL	–	Enlarged	LT4 replacement treatment Fluid drainage	–	38 weeks
Sen, Yong [9]	34	Yes	Fresh embryo transfer	Antagonis Protocol	3.84μUI/mL	13.1 pmol/l	–	–	412 IU/L	10.8 × 8.0 × 7.2 cm; 9.5 × 6.2 × 6.0 cm	LT4 replacement treatment	–	–

In these cases, the patients themselves had varying degrees of hypothyroidism. Therefore, it is necessary to use LT4 replacement therapy in advance to prevent the occurrence of HT combined with iOHSS. In one case report, although a patient with autoimmune hypothyroidism regularly took a daily dose of 0.125 mg LT4 before undergoing COH, maintaining normal serum TSH levels, she still developed OHSS after COH with an antagonist protocol [22]. In cases of autoimmune hypothyroidism or HT, using a standard dose of LT4 to maintain normal TSH levels may not effectively prevent OHSS. Even in individuals with normal TSH levels before COH, abnormal elevation of TSH and OHSS may still occur. Therefore, personalized adjustments to the dosage of LT4 are recommended for women planning to accept COH. It is advised to control the serum TSH levels of women with HT within the range of 0.27 to 2.5 mIU/L by adjusting the dosage of LT4 [22].

This preventive drug intervention may increase the pregnancy rate of these patients [59]. One patient with SHT underwent COH under the classic long protocol. She received daily dose of 0.375 mg LT4 before surgery to keep serum TSH levels below 2.5 mIU/L and daily dose of 0.05 mg LT4 from the first day of COH. Although she still developed severe OHSS on the 8th day after embryo transfer (ET), she successfully delivered with stable thyroid function by adjusting the dosage of LT4 [57]. This may be attributed to her personalized preventive LT4 replacement therapy. Another patient, with unknown subclinical autoimmune hypothyroidism, did not receive preventive LT4 replacement therapy. She developed HT and iOHSS after taking medications related to COH for 6 days to 2 weeks and failed to conceive. Considering that patients with pre-existing STH may experience worsening of their condition during or after COH and during pregnancy [60], it is particularly important to identify patients with STH early before taking medications related to COH and implement appropriate preventive measures.

In summary, patients with HT may experience iOHSS within 1 to14 days after ET. The 5 patients collected in this review who underwent COH all had thyroid disorders. For this group of patients, it is recommended to adjust dosage of LT4 before initiating COH to reduce the risk of hypothyroidism-related OHSS and other complications, as well as adverse pregnancy outcomes. Additionally, monitoring of serum TSH levels in these women should be carried out from before COH until during pregnancy. However, with only 5 reported cases, specific preventive measures and treatment options cannot be conclusively determined.

## The pathogenesis of HT combined with OHSS

**Fig. 1:**
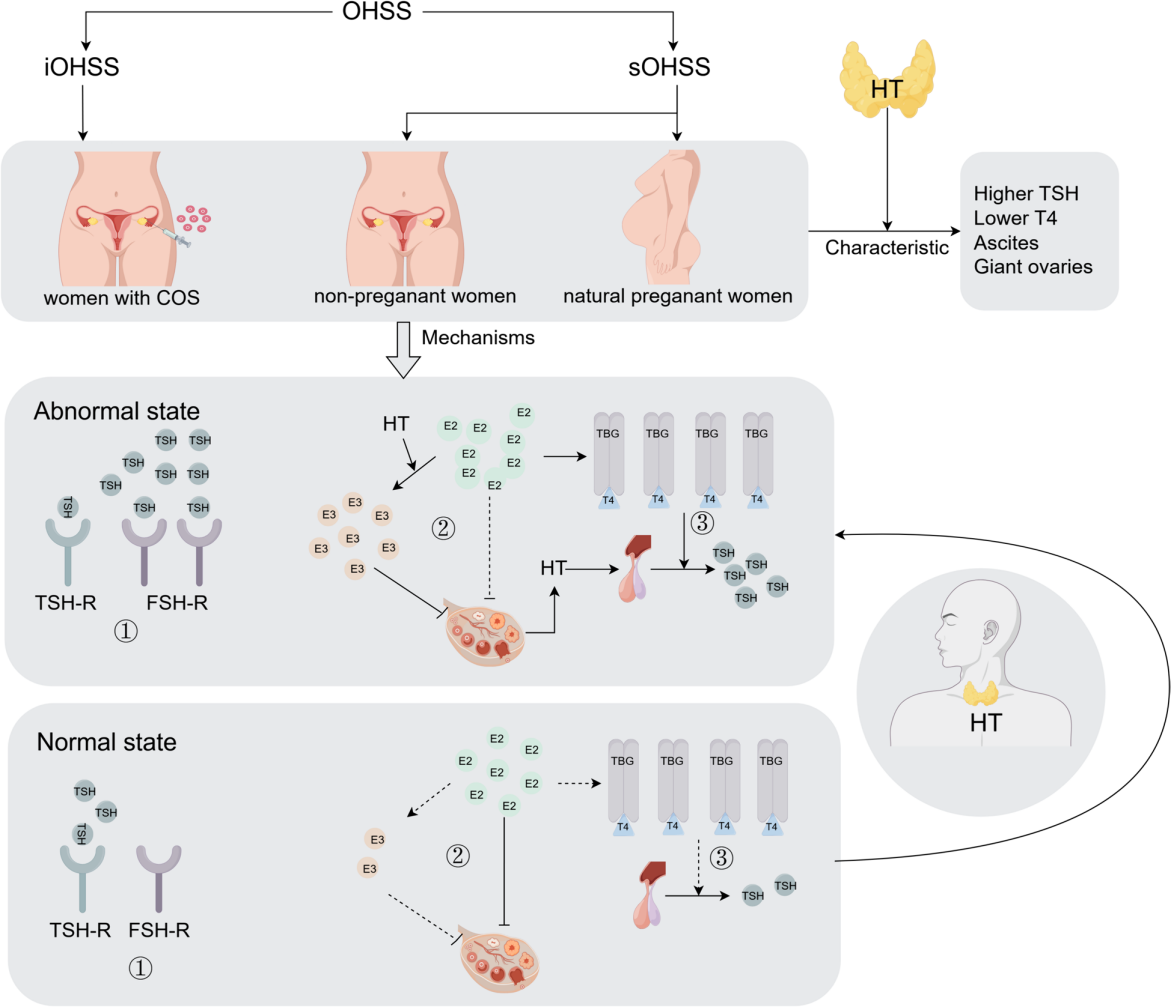
The types, population, characteristic and mechanisms of OHSS and HT. →:promoting; —|:inhibiting; ①:TSH exhibits weak FSH activity, which can activate the FSH-R; ②Patients with HT are more inclined to produce E_3_, and E_3_ has a weaker inhibitory effect on the release of gonadotropins compared to E_2_, leading to excessive release of gonadotropins; ③Elevated serum E_2_ levels can increase serum TBG levels; high levels of TBG bind more FT4, resulting in decreased serum FT4 and increased serum TSH levels

In the context of HT, the exact mechanisms underlying the occurrence of OHSS are not yet clear. Various studies have proposed the following different mechanisms: (1) Regardless of whether the FSH-R is mutated, TSH exhibits weak FSH activity, which can activate the FSH-R [61, 62]; (2) Patients with HT are more inclined to produce E_3_, and E_3_ has a weaker inhibitory effect on the release of gonadotropins hormone (GnH) compared to E_2_, leading to excessive release of gonadotropins [6, 62]; (3) Patients with HT are prone to produce more TSH, leading to activation of gonadotropin-releasing hormone receptors [31, 62]; (4) Elevated serum E_2_ levels can increase serum TBG levels; high levels of TBG bind more FT4, resulting in decreased serum FT4 and increased serum TSH levels [63]. (Fig. 1).

The FSH-R belongs to the G protein-coupled receptor family, which also includes luteinizing hormone receptor (LH-R), human chorionic gonadotropin receptor (HCG-R), and TSH-R. FSH, LH, TSH, and HCG are four hormones with similar structures [8]. They consist of two subunits, where the α subunit is common to all molecules, and the β subunit is unique to each molecule [64]. Under normal circumstances, HCG and LH bind to the LH-R, while FSH and TSH bind separately to the FSH-R and TSH-R [8, 11, 33, 65]. However, in patients with HT, low thyroid hormone levels may negatively feedback to increase the release of TSH, FSH, and LH [29, 66]. Due to the homology between the THS-R and the FSH-R, elevated serum TSH levels can cross-react with the FSH-R, leading to the occurrence of OHSS [34]. Elevated serum TSH levels may also stimulate the expression of wild-type FSH-R on the follicles. The research has shown that under conditions simulating high concentrations of TSH, TSH can activate the FSH-R and bind to it [67]. Extremely high levels of TSH binding to FSH-R lead to follicular cell activation. In bioassays, recombinant human TSH has been found to bind to FSH-R and result in a dose-dependent increase in cyclic adenosine monophosphate (cAMP) levels [68]. TSH exerts its effects through the TSHR/cAMP/protein kinase A pathway. TSH-R can be observed on the surface epithelium, primordial, primary, and secondary oocytes of the ovary [69]. One study has confirmed that there was a significant increase in cAMP concentration after 2 hours of TSH stimulation in cultured ovarian granulosa cells [70]. Elevated cAMP levels could induce granulosa cell apoptosis, which would activate PKA and then induced PI3K phosphorylation by inactivating GAP2 in granulosa cells [71]. In addition, six heterozygous activating mutations of FSH-R have been described currently. The common feature of these six mutations is their reduced specificity for FSH and responsiveness to increases in hCG or TSH concentrations. They are all located in the transmembrane, extracellular FSH-R domains and FSH-R cytoplasmic tail, associated with reduced FSH specificity [72, 73]. Mutant FSH-R might directly inhibit PI3K activation, or at least inhibit PI3K activation in the absence of cAMP involvement. PI3K is involved in granulosa cell proliferation, differentiation, survival, and enhanced mRNA translation [74].

Another possible explanation for the coexistence of HT and sOHSS may be that there is an increased activity in the 16-hydroxylation pathway in patients with HT, making it easier for E_2_ to generate higher-activity E_3_ through the 16-hydroxylation pathway instead of the normal 2-hydroxylation pathway generating lower-activity E_3_ [25, 75]. Additionally, E_3_ has a weaker inhibitory effect on gonadotropin release compared to E_2_. Therefore, the excess of higher-activity E_3_ and the reduced amount of E_2_ alleviate the negative feedback regulation of gonadotropin release, leading to excessive release of gonadotropins and the occurrence of OHSS [8, 45].

In addition, as a commonly used medication for inducing ovulation, clomiphene citrate leads to a rise in FSH concentration of about 50% with a subsequent rise in E_2_ production, some influence—a temporary lowering of fT4 concentration—on thyroid hormone levels is to be expected [59]. Patients undergoing COH experience a significant increase in serum E_2_ levels, leading to an elevation in serum TBG concentration, which may result in a decrease in serum FT4 levels and an increase in serum TSH levels [56]. The elevated levels of TSH can cross-react with FSH-R, leading to the development of delayed OHSS. In patients with PCOS, who already have elevated serum E_2_ levels, facing COH causes the excessively high concentration of serum E_2_ to rapidly stimulate the hypothalamic-pituitary-thyroid axis to produce thyroid hormones. However, the inability to rapidly produce thyroid hormones in patients with HT and PCOS, coupled with the accumulated high concentration of TSH, leads to the development of OHSS [58]. Additionally, HT may increase the risk of patients undergoing COH developing OHSS [9]. Furthermore, the activation of the cytokine signaling inhibitor and the dysregulation of IL-2 expression caused by high concentration of serum HCG are generally considered to be the causes of OHSS [76].

## Conclusion

In summary, current research on HT combined with OHSS mainly relies on case reports. Although the specific reasons for the occurrence of OHSS combined with HT vary in each case report, the typical characteristics of OHSS combined with HT mainly manifest as elevated serum TSH levels and bilateral ovarian enlargement observed in ultrasound examinations. In more severe cases, patients may also experience recurrent accumulation of ascites or pleural effusion. It is worth noting that despite slight variations in diagnostic criteria globally, the measurement of serum TSH levels and ultrasound examinations are essential means for confirming the diagnosis of this disease.

Although the diagnosis of this disease is relatively straightforward, its progression is rapid, requiring prompt and effective preventive measures. However, due to the current lack of clarity regarding the pathogenesis of the disease, it is not yet possible to predict its occurrence and development in advance. It is also challenging to fundamentally halt its progression. Despite isolated cases resorting to surgical intervention, the primary therapeutic approach currently revolves around pharmacological interventions targeting the most downstream part of the hypothalamic-pituitary-thyroid axis, involving personalized replacement therapy with oral LT4.

This study provides in-depth insights into several aspects. Firstly, considering that HT combined with OHSS is a clinical condition causing endocrine disruption, its study is crucial for optimizing patient management. The combination of HT and OHSS may lead to more complex clinical symptoms and have adverse effects on reproductive health and overall metabolism. One of the primary challenges is distinguishing HT combined with OHSS from ovarian malignancies. While both conditions may exhibit elevated serum CA125 levels and enlarged ovaries on both sides, there are significant differences in subsequent treatment approaches for these two diseases. Conscious efforts by physicians to differentiate between two diseases are essential to avoid unnecessary surgeries.

Moreover, with the ongoing advancement of ART, the number of patients planning to undergo COH is gradually increasing. Although some cases argue that routine thyroid function testing is unnecessary before COH, this review, in conjunction with several existing case reports, suggests that early thyroid function testing is necessary for infertility patients planning to accept COH. Particularly for patients with HT, a more cautious approach to personalized drug intervention is required to prevent the occurrence of OHSS or delay the progression of the disease. This can help avoid serious and life-threatening consequences associated with HT combined with OHSS. In summary, this review deepens physicians’ understanding of HT combined with OHSS, assisting in clarifying the diagnosis and treatment strategies, and improving the clinical management quality for patients with HT combined with OHSS, which holds academic and clinical value.

However, as mentioned before, the pathogenesis of this disease has not been clearly elucidated to date. Therefore, substantial progress has yet to be made in early prevention and precision medicine for this condition. Currently, there is a lack of clinical prediction models or molecular targeted markers available for predicting the risk of developing HT combined with OHSS. In the future, it may be necessary to conduct in-depth studies with large samples to develop clinical prediction models for the risk of HT combined with OHSS and explore the corresponding molecular biology mechanisms. This could prompt physicians to implement diverse, precise, and effective clinical treatment strategies for patients with HT combined with OHSS, thereby improving their quality of life and reproductive health.

## Data Availability

No datasets were generated or analysed during the current study.

## Abbreviations

AFC: Antral follicle count
ART: Assisted reproductive technology
COH: Controlled ovarian hyperstimulation
E_2_: Estradiol
E_3_: Estriol
ET: Embryo transfer
FSH: Follicle stimulating hormone
FSH-R: Follicle stimulating hormone receptors
FT4: Free thyroxine
GnH: Gonadotropins hormone
GnRH: Gonadotropin-releasing hormone
GnRH-α: Gonadotrophin-releasing hormone analogue
HCG: Human chorionic gonadotropin
HCG-R: Human chorionic gonadotropin receptors
HT: Hypothyroidism
iOHSS: Iatrogenic form of ovarian hyperstimulation syndrome
IVF: In vitro fertilization
LH: Luteinizing hormone
LH-R: Luteinizing hormone receptors
LT4: Levothyroxine
OHSS: Ovarian hyperstimulation syndrome
PCOS: Polycystic ovarian syndrome
SHT: Subclinical hypothyroidism
TBG: Thyroxine-binding globulin
TPOAb: Thyroid peroxidase antibodies
TSH: Thyroid-stimulating hormone
TSH-R: Thyroid-stimulating hormone receptors

## References

[1] Golan A, Ron-el R, Herman A, Soffer Y, Weinraub Z, Caspi E (1989). Ovarian hyperstimulation syndrome: an update review. Obstet Gynecol Surv.

[2] Whelan JG, Vlahos NF (2000). The ovarian hyperstimulation syndrome. Fertil Steril.

[3] Daolio J, Sperduti S, Casarini L, Falbo A, Materazzo C, Aguzzoli L (2023). Spontaneous and iatrogenic ovarian hyperstimulation syndrome in the absence of FSHR mutations: a case report of two unexpected cases. BMC Med Genet.

[4] Ovarian hyperstimulation syndrome. Fertil Steril. 2008;90(5 Suppl):S188–93. 10.1016/j.fertnstert.2008.08.034 19007627

[5] Delbaere A, Smits G, Olatunbosun O, Pierson R, Vassart G, Costagliola S (2004). New insights into the pathophysiology of ovarian hyperstimulation syndrome. What makes the difference between spontaneous and iatrogenic syndrome?. Hum Reprod (Oxford, England)..

[6] Kanza RE, Gagnon S, Villeneuve H, Laverdiere D, Rousseau I, Bordeleau E (2013). Spontaneous ovarian hyperstimulation syndrome and pituitary hyperplasia mimicking macroadenoma associated with primary hypothyroidism. World J Radiol.

[7] Nwafor NN, Nyoyoko NP (2020). Spontaneous ovarian Hyperstimulation syndrome: a report of two cases from different pathogenesis. Niger Med J : J Niger Med Assoc.

[8] Pail S, Bagri N, Ghas R (2023). Hypothyroidism induced spontaneous ovarian hyperstimulation syndrome: a rare yet interesting dilemma. New Indian J OBGYN.

[9] Sen S, Yong TT, Yu SL, Rajesh H (2019). Isolated unilateral pleural effusion without ascites in late onset ovarian Hyperstimulation syndrome: a case report and review of literature. Fertil Reprod.

[10] Zheng D, Shi Y, Wang Y, Li R, Long X, Qiao J (2023). The incidence of moderate and severe ovarian hyperstimulation syndrome in hospitalized patients in China. Health Data Sci..

[11] Chae H-D, Park E-J, Kim S-H, Kim C-H, Kang B-M, Chang YS (2001). Case report: ovarian hyperstimulation syndrome complicating a spontaneous singleton pregnancy: a case report. J Assist Reprod Genet.

[12] Lewis CG, Warnes GM, Wang XJ, Matthews CD (1990). Failure of body mass index or body weight to influence markedly the response to ovarian hyperstimulation in normal cycling women. Fertil Steril.

[13] Rahimianfar F, Rahimianfar M, Eshghjoo S (2023). The varied impacts of vascular endothelial growth factor-a (VEGF-a) on vasculature in ovarian hyper stimulation syndrome (OHSS) and obesity.

[14] Corbett S, Shmorgun D, Claman P (2014). The prevention of ovarian hyperstimulation syndrome. J Obstet Gynaecol Can : JOGC = Journal d'obstetrique et gynecologie du Canada : JOGC.

[15] Monaco F (2003). Classification of thyroid diseases: suggestions for a revision. J Clin Endocrinol Metabol.

[16] Bachmakova NV, Dubrovina OS, Lisovskaya TV, Melkozerova OA, Maysina EN, Sentiurina LB (2014). The development of ovarian hyperstimulation syndrome in the implementation of assisted reproductive technology in women with a background of endocrine pathology. Gynecol Endocrinol.

[17] Busnelli A, Cirillo F, Levi-Setti PE (2021). Thyroid function modifications in women undergoing controlled ovarian hyperstimulation for in vitro fertilization: a systematic review and meta-analysis. Fertil Steril.

[18] Ashrafi M, Bahmanabadi A, Akhond MR, Arabipoor A (2015). Predictive factors of early moderate/severe ovarian hyperstimulation syndrome in non-polycystic ovarian syndrome patients: a statistical model. Arch Gynecol Obstet.

[19] Muller AF, Verhoeff A, Mantel MJ, de Jong FH, Berghout A (2000). Decrease of free thyroxine levels after controlled ovarian Hyperstimulation1. J Clin Endocrinol Metabol.

[20] Mintziori G, Goulis DG, Toulis KA, Venetis CA, Kolibianakis EM, Tarlatzis BC (2011). Thyroid function during ovarian stimulation: a systematic review. Fertil Steril.

[21] Du Y, Xin X, Cui N, Jiang L, Yang A, Hao G (2019). Effects of controlled ovarian stimulation on thyroid stimulating hormone in infertile women. Eur J Obstet Gynecol Reprod Biol.

[22] Poppe K, Glinoer D, Tournaye H, Devroey P, Velkeniers B (2008). Impact of the ovarian hyperstimulation syndrome on thyroid function. Thyroid : Off J Am Thyroid Assoc.

[23] Poppe K, Unuane D, D'Haeseleer M, Tournaye H, Schiettecatte J, Haentjens P (2011). Thyroid function after controlled ovarian hyperstimulation in women with and without the hyperstimulation syndrome. Fertil Steril.

[24] Pekonen F, Alfthan H, Stenman ULFH, Ylikorkala O (1988). Human chorionic gonadotropin (hCG) and thyroid function in early human pregnancy: circadian variation and evidence for intrinsic Thyrotropic activity of hCG*. J Clin Endocrinol Metabol.

[25] Rotmensch S, Scommegna A (1989). Spontaneous ovarian hyperstimulation syndrome associated with hypothyroidism. Am J Obstet Gynecol.

[26] Hedayati Emami M, Molaei Langroudi R, Ghazanfari AF (2012). Ovarian hyperstimulation syndrome and autoimmune primary hypothyroidism in two members of a family. J Clin Case Rep.

[27] Langroudi RM, Amlashi FG, Emami MHH. Ovarian cyst regression with levothyroxine in ovarian hyperstimulation syndrome associated with hypothyroidism. Endocrinol Diabetes Metab Case Rep. 2013;2013(1). 10.1530/EDM-13-0006 PMC3922305 24616758

[28] Chen CP, Chen CW, Wang KG (1996). Spontaneous ovarian hyperstimulation syndrome and hyperprolactinemia in primary hypothyroidism. Acta Obstet Gynecol Scand.

[29] Taher BM, Ghariabeh RA, Jarrah NS, Hadidy AM, Radaideh AM, Ajlouni KM (2004). Spontaneous ovarian hyperstimulation syndrome caused by hypothyroidism in an adult. Eur J Obstet Gynecol Reprod Biol.

[30] PKaluarachchi D, Casather D, Rathnayaka R, Ramachandran R, Herath R, Mettananda S. Spontaneous ovarian hyperstimulation syndrome as a presenting manifestation of acquired hypothyroidism. Sri Lanka Journal of Child Health. 2023:53.

[31] Putta T, John R, Thomas N, Jebasingh F, Peedicayil A, Eapen A (2016). A case of spontaneous ovarian hyperstimulation syndrome (SOHSS) due to hypothyroidism. Aust Med J (Online).

[32] Rajaram S, Bhaskaran S, Aggarwal P, Goel N (2015). Spontaneous ovarian hyperstimulation mimicking ovarian neoplasm: a rare complication of hypothyroidism. J Obstet Gynaecol : J Inst Obstet Gynaecol.

[33] Ilanchezhian S, Mohan SV, Ramachandran R, Babu SR (2015). Spontaneous ovarian hyperstimulation syndrome with primary hypothyroidism: imaging a rare entity. Radiol Case Rep.

[34] Guvenal F, Guvenal T, Timuroglu Y, Timuroglu T, Cetin M (2006). Spontaneous ovarian hyperstimulation-like reaction caused by primary hypothyroidism. Acta Obstet Gynecol Scand.

[35] Van Voorhis BJ, Neff TW, Syrop CH, Chapler FK (1994). Primary hypothyroidism associated with multicystic ovaries and ovarian torsion in an adult. Obstet Gynecol.

[36] Katulande P, Kariyawasam SS, Senanayake HM, Weerakkodi M (2013). Multicystic ovaries and pituitary pseudo-adenoma associated with primary hypothyroidism. J Obstet Gynaecol.

[37] Erol O, Erol MB, Ayik H, Derbent AU (2013). Recurrent spontaneous ovarian hyperstimulation secondary to hypothyroidism in an adolescent girl. Arch Gynecol Obstet.

[38] Singh A, Singh K, Khandelwal RG, Choudhary P, Sharma VK (2015). Spontaneous severe ovarian hyper stimulation syndrome associated with massive pericardial effusion and hypothyroidism in non-pregnant woman. J Obstet Gynecol India.

[39] Kim SJ, Yoon JH, Kim HK, Kang HC (2017). Spontaneous ovarian hyperstimulation syndrome in a young female subject with a lingual thyroid and primary hypothyroidism. Korean J Intern Med.

[40] Patel S, Nath P (2020). Spontaneous ovarian Hyperstimulation syndrome: looking beyond the ovary. Fertil Reprod.

[41] Emami MHH, Langroudi RM, Amlashi FG. Ovarian Hyperstimulation syndrome and autoimmune primary hypothyroidism in two members of a family. J Clin Case Rep. 2012;2012.

[42] Edwards-Silva R, Han C, Hoang Y, Kao L-C (2008). Spontaneous ovarian Hyperstimulation in a naturally conceived pregnancy with uncontrolled hypothyroidism. Obstet Gynecol.

[43] Dieterich M, Bolz M, Reimer T, Costagliola S, Gerber B (2010). Two different entities of spontaneous ovarian hyperstimulation in a woman with FSH receptor mutation. Reprod Biomed Online.

[44] Nappi RG, Di Naro E, D'Aries AP, Nappi L (1998). Natural pregnancy in hypothyroid woman complicated by spontaneous ovarian hyperstimulation syndrome. Am J Obstet Gynecol.

[45] Cardoso CG, Graça LM, Dias T, Clode N, Soares L (1999). Spontaneous ovarian hyperstimulation and primary hypothyroidism with a naturally conceived pregnancy. Obstet Gynecol.

[46] Lussiana C, Guani B, Restagno G, Rovei V, Menato G, Revelli A (2009). Ovarian hyper-stimulation syndrome after spontaneous conception. Gynecol Endocrinol.

[47] Borna S, Nasery A (2007). Spontaneous ovarian hyperstimulation in a pregnant woman with hypothyroidism. Fertil Steril.

[48] Akbay E, Uzunçakmak C, İdil NS, Akçiğ Z, Özel G, Yaşar L (2010). Recurrent spontaneous ovarian hyperstimulation syndrome with hypothyroidism: a case report. Bakırköy Tıp Dergisi.

[49] Delabaere A, Tran X, Jardon K, Pouly JL, Bourdel N (2011). Spontaneous ovarian hyperstimulation syndrome in a pregnancy with hypothyroidism. Gynecol Obstet Fertil.

[50] Sridev S, Barathan S (2013). Case report on spontaneous ovarian hyperstimulation syndrome following natural conception associated with primary hypothyroidism. J Hum Reprod Sci.

[51] Seethapathy DK. Spontaneous ovarian HYPERSTIMULATION in a naturally conceived pregnancy with uncontrolled hypothyroidism-a rare CASE report. Univ J Surg Surg Specialities. 2018;4(4).

[52] Oliveira ESL, Innecco Arêas JV, Rezende Campos MC, Innecco Arêas I, Martins Resende BA (2021). Spontaneous ovarian hyperstimulation syndrome in a pregnant woman with hypothyroidism: a case report. FS Rep.

[53] Alzebidi JA, Almushri K, Elmoheen R, Alzebidi J. Spontaneous ovarian Hyperstimulation syndrome associated with primary hypothyroidism. Cureus. 2023;15(1). 10.7759/cureus.33247 PMC9808876 36606102

[54] Guerra M, Marado D, Silva F, Almeida MC. Severe primary hypothyroidism and ovarian hyperstimulation syndrome in a spontaneous pregnancy: a case report. AME Case Rep. 2023;8. 10.21037/acr-23-13 PMC10789883 38234339

[55] Aiura R, Nakayama S, Yamaga H, Kato Y, Fujishima H (2021). Systemic thromboembolism including multiple cerebral infarctions with middle cerebral artery occlusion caused by the progression of adenomyosis with benign gynecological tumor: a case report. BMC Neurol.

[56] Ghianda LE, Ruggiero M, Benelli E, Artini PG, Cela V (2014). Over hypothyroidism in a woman undergoing controlled ovarian Hyperstimulation. Endocr Pract.

[57] Skweres T, Wójcik D, Ciepłuch R, Sliwiński W, Czech R, Gruszczyński W (2014). Thyroid dysfunction during severe ovarian hyperstimulation syndrome. A case report. Ginekol Pol.

[58] Galvao A, Lourenço C, Fraga S, Pereira I, Barreiro M (2018). Bilateral massive vulvar edema and thyroid dysfunction in ovarian hyperstimulation syndrome. Acta Obstet Ginecol Port.

[59] Muller AF (2016). Other endocrine disorders causing anovulation: thyroid disorders. Ovulation Induction.

[60] Lee Y, Kim C, Kwack J, Ahn J, Kim S, Chae H (2014). Subclinical hypothyroidism diagnosed by thyrotropin-releasing hormone stimulation test in infertile women with basal thyroid-stimulating hormone levels of 2.5 to 5.0 mIU/L. Ogs..

[61] Kasum M, Oresković S (2011). New insights in prediction of ovarian hyperstimulation syndrome. Acta Clin Croat.

[62] Sultan A, Velaga MR, Fleet M, Cheetham T (2006). Cullen's sign and massive ovarian enlargement secondary to primary hypothyroidism in a patient with a normal FSH receptor. Arch Dis Child.

[63] Fleischer K, Muller A, Hohmann F, de Jong F, Eijkemans R, Fauser B (2014). Impact of controlled ovarian hyperstimulation on thyroid function. Reprod Biol Insights.

[64] Rodien P, Beau I, Vasseur C (2010). Ovarian hyperstimulation syndrome (OHSS) due to mutations in the follicle-stimulating hormone receptor. Annales d'endocrinologie.

[65] Ebru A, Cihangir U, Idil N, Zeynep A, Gül Ö, Yaşar L. Recurrent spontaneous ovarian hyperstimulation syndrome with hypothyroidism: a case report. Med J Bakirköy. 2010;6.

[66] Grumbach M, Styne D, Wilson JD, Foster DW, Kronenberg HM, Larsen PR (1998). Puberty: ontogeny, neuroendocrinology, physiology, and disorders. Williams textbook of endocrinology.

[67] Kasum M, Oresković S, Jezek D (2013). Spontaneous ovarian hyperstimulation syndrome. Coll Antropol.

[68] Shivaprasad K, Dutta D, Jain R, Kumar M, Maisnam I, Biswas D (2013). Huge bilateral ovarian cysts in adulthood as the presenting feature of Van Wyk Grumbach syndrome due to chronic uncontrolled juvenile hypothyroidism. Indian J Endocrinol Metab.

[69] Aghajanova L, Lindeberg M, Carlsson IB, Stavreus-Evers A, Zhang P, Scott JE (2009). Receptors for thyroid-stimulating hormone and thyroid hormones in human ovarian tissue. Reprod Biomed Online.

[70] Gao H, Lu X, Huang H, Ji H, Zhang L, Su Z (2021). Thyroid-stimulating hormone level is negatively associated with fertilization rate in patients with polycystic ovary syndrome undergoing in vitro fertilization. Int J Gynecol Obstet.

[71] Hunzicker-Dunn ME, Lopez-Biladeau B, Law NC, Fiedler SE, Carr DW, Maizels ET (2012). PKA and GAB2 play central roles in the FSH signaling pathway to PI3K and AKT in ovarian granulosa cells. Proc Natl Acad Sci.

[72] Hugon-Rodin J, Sonigo C, Gompel A, Dodé C, Grynberg M, Binart N (2017). First mutation in the FSHR cytoplasmic tail identified in a non-pregnant woman with spontaneous ovarian hyperstimulation syndrome. BMC Med Genet..

[73] Di Carlo C, Savoia F, Fabozzi A, Gargano V, Nappi C (2015). A case of ovarian torsion in a patient carrier of a FSH receptor gene mutation previously affected by spontaneous ovarian hyperstimulation syndrome. Gynecol Endocrinol.

[74] Uchida S, Uchida H, Maruyama T, Kajitani T, Oda H, Miyazaki K (2013). Molecular analysis of a mutated FSH receptor detected in a patient with spontaneous ovarian hyperstimulation syndrome. PLoS One.

[75] Raju UMA, Bradlow HL, Levitz M (1990). Estriol-3-sulfate in human breast cyst Fluida. Ann N Y Acad Sci.

[76] Mirzaeian S, Jafari M, Jafarzadeh R (2019). Spontaneous ovarian hyperstimulation syndrome in second pregnancy of a healthy pregnant woman. Acta Med Iran.

